# Identification of tumor antigens and immune subtypes in lower grade gliomas for mRNA vaccine development

**DOI:** 10.1186/s12967-021-03014-x

**Published:** 2021-08-17

**Authors:** Liguo Ye, Long Wang, Ji’an Yang, Ping Hu, Chunyu Zhang, Shi’ao Tong, Zhennan Liu, Daofeng Tian

**Affiliations:** Department of Neurosurgery, Renmin Hospital of Wuhan University, Wuhan, 430060 Hubei Province P.R. China

**Keywords:** Lower grade glioma, Tumor antigens, Immunotyping, Cancer vaccination, Bioinformatics

## Abstract

**Background:**

As an important part of tumor immunotherapy for adjunct, therapeutic tumor vaccines have been effective against multiple solid cancers, while their efficacy against lower grade glioma (LGG) remains undefined. Immunophenotyping of tumors is an essential tool to evaluate the immune function of patients with immunodeficiency or autoimmunity. Therefore, this study aims to find the potential tumor antigen of LGG and identify the suitable population for cancer vaccination based on the immune landscape.

**Method:**

The genomic and clinical data of 529 patients with LGG were obtained from TCGA, the mRNA_seq data of normal brain tissue were downloaded from GTEx. Differential expression gene and mutation analysis were performed to screen out potential antigens, K-M curves were carried out to investigate the correlation between the level of potential antigens and OS and DFS of patients. TIMER dataset was used to explore the correlation between genes and immune infiltrating cells. Immunophenotyping of 529 tumor samples was based on the single-sample gene sets enrichment analysis. Cibersort and Estimate algorithm were used to explore the tumor immune microenvironment characteristics in each immune subtype. Weighted gene co-expression network analysis (WGCNA) clustered immune-related genes and screened the hub genes, and pathway enrichment analyses were performed on the hub modules related to immune subtype in the WGCNA.

**Results:**

Selecting for the mutated, up-regulated, prognosis- and immune-related genes, four potential tumor antigens were identified in LGG. They were also significantly positively associated with the antigen-presenting immune cells (APCs). Three robust immune subtypes, IS1, IS2 and IS3, represented immune status "desert", "immune inhibition", and "inflamed" respectively, which might serve as a predictive parameter. Subsequently, clinicopathological features, including the codeletion status of 1p19q, IDH mutation status, tumor mutation burden, tumor stemness, etc., were significantly different among subtypes.

**Conclusion:**

FCGBP, FLNC, TLR7, and CSF2RA were potential antigens for developing cancer vaccination, and the patients in IS3 were considered the most suitable for vaccination in LGG.

**Supplementary Information:**

The online version contains supplementary material available at 10.1186/s12967-021-03014-x.

## Introduction

Gliomas were the most common human primary central nervous tumor, and lower grade gliomas (LGG), including World Health Organization (WHO) II, III grade, compose the largest subgroup in all gliomas [1, 2]. At present, the primary available treatment for LGG is still surgical resection. However, due to the silent clinical characteristics of LGG, most patients miss the suitable opportunity to for surgery [3]. Besides, the combination of radiotherapy and temozolomide chemotherapy is the first-line adjuvant strategy that could increase the patients'' survival time by 2.5 months [4] but still with a high risk of acquired primary resistance [3]. Hence, novel strategies are needed to improve the therapeutic condition of LGG. Nowadays, as an important part of tumor immunotherapy, therapeutic tumor vaccines were recently reported to be effective against multiple solid cancers and have attracted extensive attention [5], while its efficacy against LGG remains undefined. Moreover, identifying a growing number of potentially unique immunoreactive tumor-associated antigens expressed by human gliomas makes cancer vaccines an exciting strategy [6].

Tumor antigen with or without adjuvant is the main component of a typical cancer vaccine, assisting immune cells in recognizing and eliminating cancer cells [7]. The advantages were minimal non-specific effect, non-toxic, long-term immune memory and wide treatment window for tumor vaccine treatment which could overcome the limits of drug resistance, high costs, limited therapeutic effects and other possible adverse reactions associated with traditional immunotherapy and chemotherapy [8]. The form of antigens for tumor vaccine could be peptide, tumor cell, dendritic cell, DNA, and RNA type [9]. However, when applied in clinical treatment, there were several prominent advantages for mRNA type compared with the first four types. First of all, the mRNA sequence can be easily modified to encode the protein we need [10]. Second, genetic analysis of cancer was required in the traditional peptide vaccine which needs a relatively high cost, while mRNA vaccine does not need [11]. Third, to ensure safety, the half-life of mRNA could be regulated through RNA sequence modification or a delivery system [12]. Fourth, preventing gene deletion and insertional mutagenesis, mRNA has no risk of irrelevant sequence exclusion and gene integration which often happen to DNA type [13]. In addition, increasing its in-vivo immunogenicity, the adjuvant properties of mRNA vaccine could induce an intense and persistent immune response [14]. As a result, mRNA vaccines are highly feasible for targeting tumor-specific antigens and promising immunotherapy strategies. Several studies have proved the effectiveness of the possibility of mRNA tumor vaccines in clinical trials, Sebastian et al. [15] reported that the RNActive® vaccine CV9201 could improve the specific immune response rate and survival time of a part of patients with non-small cell lung cancer. Similarly, the study of Kübler et al. [16] showed that CV9103 can maintain well immunogenicity and tolerance in a large part of prostate cancer patients, enhancing the immune response of patients and prolong the overall survival time ultimately. However, for patients with LGG, no specific mRNA vaccine against tumor has been developed and no study have identified suitable patients for cancer vaccination based on immunophenotyping.

In our study, four candidates identified for developing mRNA vaccines were associated with clinical outcomes and positively correlated to the infiltration of antigen-presenting cells (APCs). Based on the clustering of immune-related differently expressed genes (IRDEGs), three robust immune subtypes were identified based on the features of TIME in each subtype. We then screened three functional modules closely related to subtypes through WGCNA. These findings provided a theoretical basis for developing mRNA cancer vaccine against LGG, described an immune landscape and identified candidate population for mRNA cancer vaccination.

## Methods

### Data acquisition

The normalized gene expression and corresponding clinical follow-up data of 529 LGG patients were downloaded from The Cancer Genome Atlas (TCGA). Furthermore, the mRNA data of 940 normal brain tissue samples were obtained from Genotype-Tissue Expression (GTEx) project. Then the mRNA data in TCGA and GTEx were merged and normalized as one cohort by R package "limma".

The data of simple nucleotide variation, including somatic mutation according to the VarScan2 [17] platform, were acquired from TCGA.

### Patient samples

The Institutional Ethics Committee approved this study of the Faculty of Medicine at our hospital. Informed consent was obtained from all patients whose tissues were used. In total, 6 control samples from patients with cerebral hemorrhage and 24 lower-grade glioma samples (WHO grade II-III) were collected during May 2019 and June 2021. All patients were not treated with chemotherapy or radiotherapy before surgery.

### Data processing

R package "maftools" was used to identify the mutant genes in LGG and the corresponding chromosome position of genes. Over-expressed genes in the tumor were identified in the merged cohort by "limma" package based on the criterion: the ABS of logFC > 1 and p value < 0.05. By "estimate" algorithm, the immune infiltration level of each tumor sample was calculated and quantified as stromal score and immune score. According to the median value of stromal and immune scores, respectively, the samples were divided into high and low score groups, genes differentially expressed in two groups were screened by "limma" package and defined as immune-related differentially expressed genes (IRDEGs). The intersection of mutant genes, overexpressed genes, and IRDEGs was considered the potential mRNA cancer antigens in LGG.

### Prognostic analysis of potential antigens

Kaplan–Meier (K–M) survival analysis was performed to explore the relationship between potential antigens’ expression level and overall survival (OS) rate in patients. Then based on the Gene Expression Profiling Interactive Analysis (GEPIA) database, the relationship between genes and disease-free survival (DFS) of LGG patients was investigated, log-rank P-value < 0.05 was considered significant.

### TIMER analysis

Tumor Immune Estimation Resource [18] (TIMER) was used to analyze and visualize the association between the abundance of tumor immune infiltrating cells (TIICs) and prognosis-related antigens. Considering purity adjustment, the relationship between potential LGG antigens and antigen-presenting cells (APCs), including B cells, macrophages, and dendritic cells, was investigated through spearman's correlation analysis. P-value < 0.05 was significant.

### Quantitative real-time PCR

The extraction of potential LGG antigens' RNA from tissues and cells was carried out by Trizol reagent (Invitrogen, Carlsbad, CA, USA). The PrimeScript RT Reagent Kit (RR047A, Takara, Japan) was used to synthesize cDNA. We used SYBR Premix Ex Taq II (RR820A, Takara, Kusatsu, Japan) and Bio-Rad CFX Manager 2.1 real-time PCR Systems (Bio-Rad, Hercules, CA, USA) to detect mRNA levels following the specifications provided by the manufacturers. Adopt the relative Ct method to compare the data of the experimental group and the control group, and GADPH was set as an internal control.

### Development and validation of the immune subtypes

The 1113 IRDEGs were clustered based on their expression profiles, and a consistency matrix was constructed to identify corresponding immune subtypes. The partition around medoids algorithm using the "1-Pearson correlation" distance metric was applied, and 500 bootstraps were performed, each involving 80% patients in the discovery cohort. Cluster sets varied from 2 to 9, and the optimal partition was defined by evaluating the consensus matrix and the consensus cumulative distribution function. Besides, the correlation between immune subtypes and clinical features, molecular subtypes, tumor mutation burden (TMB), and tumor stemness indices were explored to describe the clinical and molecular pathological features among immune subtypes we defined.

### The ssGSEA of immune subtypes

In the TCGA dataset, 29 immune signatures[19] representing diverse immune cell types, functions, and pathways were quantified for their enrichment degrees within respective LGG samples using single sample gene set enrichment analysis (ssGSEA)[20]. The ssGSEA score of each LGG sample was calculated and then compared among different immune subtypes.

### TIICs profiles in different subtypes

Through "cibersort" [21] algorithm, the abundance of TIICs in each LGG sample were evaluated and then compared among subgroups, exploring the features of tumor immune microenvironment (TIME) in each immune subtype.

### Differential expression analysis of ICPs and ICDs

Immune checkpoints (ICPs)- and immunogenic cell death modulators (ICDs)-related genes were obtained from the previous studies[7, 22]. Then the expression level of ICPs and ICDs were compared among different immune subtypes by Pairwise t-tests[23].

### Weight gene co-expression network analysis

The R package "WGCNA" was used to identify the co-expression modules of the IRDEGs. Highly variable genes of HPC population were detected by FindVariableGenes in Seurat. Gene modules were examined by dynamic hybrid cut. The relationship between module genes and immune subtypes was investigated (P-value < 0.05 were considered significant). Gene Ontology (GO) and Kyoto Encyclopedia of Genes and Genomes (KEGG) analysis were used to annotate the functions of the modules correlated to immune subtypes.

## Results

### Identification of potential tumor antigens of LGG

**Fig. 1:**
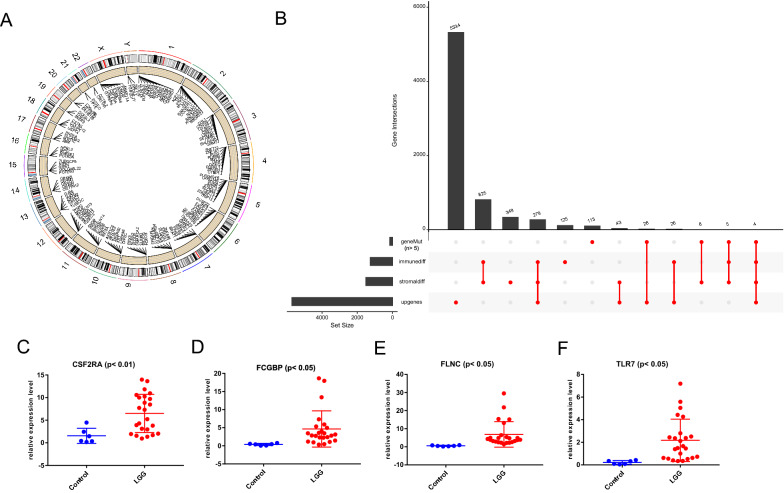
Identification of potential LGG tumor vaccine mRNA antigens. **A** The chromosomal distribution of the mutant genes in LGG. **B** The number of mutant genes, differentially expressed genes in the stromal and immune score, and up-regulated genes in LGG is shown. **C**–**F** The rt-PCR results showed the relative expression level of CSF2RA, FCGBP, FLNC, and TLR7 among control and LGG tissues. LGG: lower grade glioma; geneMut: mutant genes; immunediff: differentially expressed genes among different immune score groups; stromaldiff: differentially expressed genes among different stromal score groups; upgenes: up-regulated genes in LGG

First, mutant genes (the number of mutations in LGG samples was more than 5) in LGG were selected, and their corresponding positions in the human chromosome were shown in Fig. 1A. Then, 1,113 IRDEGs were obtained according to the intersecting of stromal (1513 genes) and immune score (1,264 genes) related DEGs. Subsequently, up-regulated genes were screened out from differentially expressed analysis among glioma and normal tissues. Finally, four potential antigens, FCGBP, FLNC, TLR7, and CSF2RA, were identified through the intersection of overexpressed genes, mutant genes, and IRDEGs. The number of each term above was displayed in the plot (Fig. 1B). The mutation landscape in LGG was shown in the figure S1 (Additional file 1).

Then we detected the samples collected in our hospital, the control and four potential antigens' primer sequences are as following: GAPDH 5′-GGAGCGAGATCCCTCCAAAAT-3′(Forward), 5′-GGCTG TTGTCATACTTCTCATGG-3′(Reverse), CSF2RA 5’-TGCTCTTCTCCACGCTACTG-3' (Forward), 5'- GGGGTCGAAGGTCAGGTTG-3' (Reverse), FCGBP 5'- GCCAAGGCTGAGATGATAGGC-3' (Forward), 5'- CCTGCACAGAGATGGCATAGT-3' (Reverse), FLNC 5'- CTGGGCGATGAGACAGACG-3' (Forward), 5'- GCGGATGGAACTTGCGGTA-3' (Reverse), and TLR7 5′-TCCTTGGGGCTAGATGGTTTC-3′(Forward), 5′-TCCACGATCACATGGTTCTTTG-3′(Reverse). We found that the level of these four genes were both significantly over-expressed in LGG compared to control brain groups (Fig. 1C–F).

### Prognostic value of four tumor antigens in LGG

**Fig. 2:**
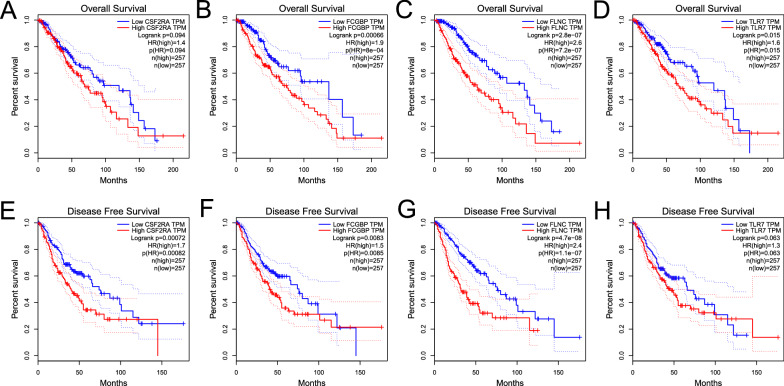
The prognostic value of four potential antigens. According to the GEPIA database, the K-M curves showed the OS of patients with LGG in the different expression levels of **A** CSF2RA, **B** FCGBP, **C** FLNC, and **D** TLR7. The correlation between DFS and **E** CSF2RA, **F** FCGBP, **G** FLNC, and **H** TLR7

As the Fig. 2A–D showed, except for CSF2RA (p > 0.05), the high expression level of FCGBP, FLNC, and TLR7 were significantly correlated to the more inferior OS of patients. While there was no significant difference between the level of TLR7 expression and DFS (Fig. 2E–H). It suggested that the potential tumor antigen identified in this work is related to the prognosis of patients with LGG.

### The expression of potential antigens was positively correlated with APCs

**Fig. 3:**
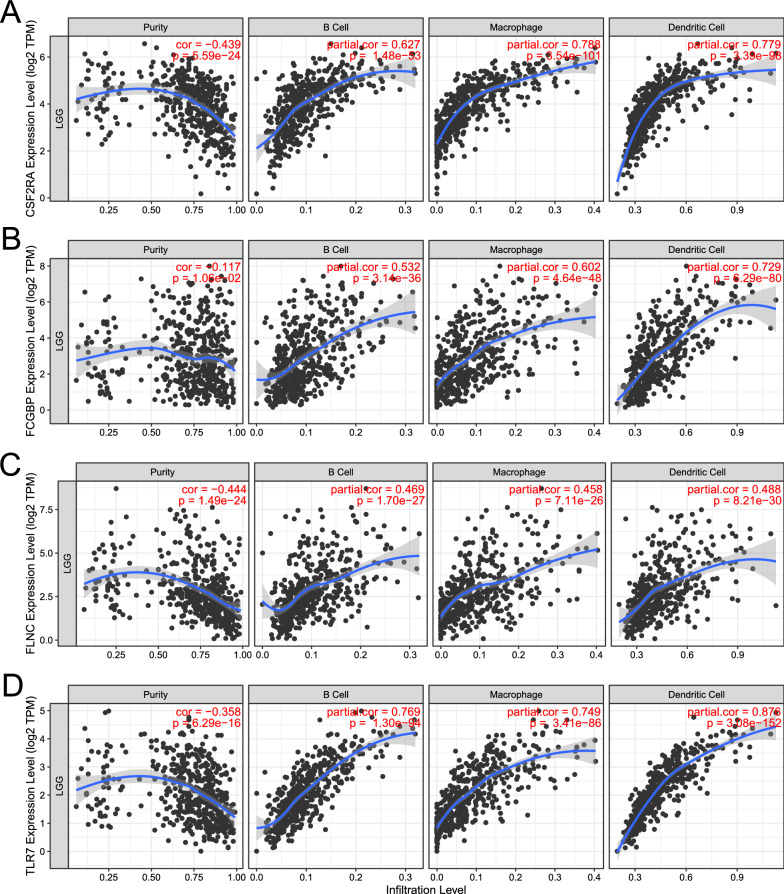
The association between four potential LGG antigens and APCs. According to the TIMER database, the correlation between tumor purity, the infiltration level of APCs (B cell, Macrophages, and DC cells) and the level of **A** CSF2RA, **B** FCGBP, **C** FLNC, and **D** TLR7

Antigen-presenting cells (APCs) play a major role in the onset of protective immunity [24]. Dendritic cells are central to initiating, regulating, and maintaining immune responses while also playing an essential role in inducing anti-tumor immune responses [25]. The role of B cells as APCs has been extensively studied, mainly about activating memory T cells and initiating APCs [26]. As shown in Fig. 3A–D, based on the TIMER algorithm, the infiltration level of APCs is significantly positively correlated with the expression level of 4 potential antigens. These findings suggest that the identified tumor antigens, processed and presented by the APCs, could trigger an better immune response. Therefore, CSF2RA, FCGBP, FLNC, and TLR7 were promising candidates for developing mRNA vaccines against LGG.

### Identification of molecular subtypes of LGG

**Fig. 4:**
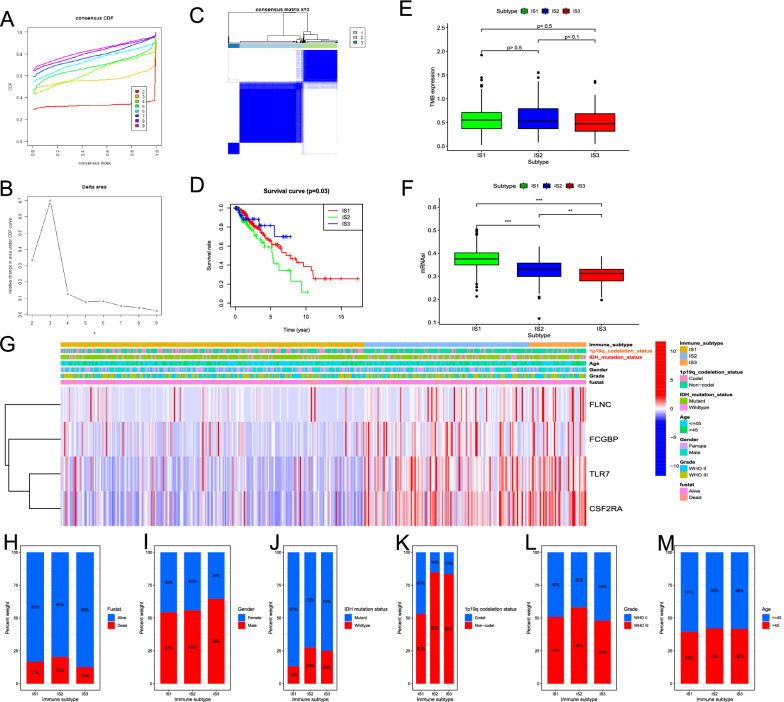
Identification of immune subtypes of LGG based on the expression of IRDEGs. **A** Consensus clustering CDF for k = 2 to k = 9. **B** Relative change in area under CDF curve for k = 2 to k = 9. **C** Consensus clustering matrix of 529 TCGA-LGG samples for k = 3. **D** Survival analysis between OS and three groups. **E** The difference of TMB changes between IS1, IS2, and IS3. **F** The difference analysis of mRNAsi on different groups. **G** Difference analysis of clinicopathological characteristics and expression level of OS-related potential LGG antigens in different subgroups. Distribution ratio of IS1-IS3 across LGG **H** fustat, **I** gender, **J** IDH mutation status, **K** 1p19q co-deletion status, **L** grade and **M** age groups (> 45 vs. <  = 45) in TCGA-LGG. Fustat, survival status

Based on the expression of IRDEGs in LGG, molecular typing data are categorized into three groups which were defined as immune subtype 1 (IS1), immune subtype 2 (IS2), and immune subtype 3 (IS3) according to the corresponding cumulative distribution function and function delta area of K value (Fig. 4A, B), IRDEGs appeared to be stably clustered when k = 3 (Fig. 4C). Survival analysis in Fig. 4D showed a significant difference between subtypes, in which the samples in IS2 had the worse OS, instead, the patients in IS3 tend to have the best clinical outcome, and IS1 was in between. We further investigated the tumor mutation burden (TMB) in the three subtypes and found no significant difference among different subtypes (Fig. 4E). Cancer stem cell characteristics are correlated to enhanced cell invasiveness, and the stem cell-associated indices, such as mRNAsi, could quantify the cancer stemness of tumor samples. We found that the mRNAsi score in IS1 was higher than IS2 and IS3 (Fig. 4F), which suggested samples in IS1, with a higher self-renewal capacity, tumorigenicity, metastatic potential, tumor-initiating ability, and chemoresistance than in other immune subtypes [27], had higher possibility of transforming into more malignant gliomas. Moreover, the clinicopathological characteristics and the expression level of potential tumor antigens were compared among three subtypes (Fig. 4G). The samples with higher levels of FLNC, FCGBP, TLR7, and CSF2RA were more found in the IS3 and IS2, which indicated patients in these subtypes may have higher specificity for mRNA vaccine therapy in LGG. Figure 4H–M displayed the percent weight of proportion for different clinicopathological subtypes in IS1, IS2, and IS3, respectively. The proportion of 1q19q co-deletion and IDH mutant status was significantly higher in IS1 than IS2 and IS3. These results indicated that patients in IS1 had higher possibility of neoplastic progression, tumor recurrence, and metastasis in LGG, while IS2 and IS3 may have higher specificity for tumor vaccination. However, it is puzzling that IS2 and IS3 have almost similar clinicopathological features, but the prognostic outcome of the groups was utterly different, which needs further analysis in TIME.

### Characteristics of TIME in different subtypes

**Fig. 5:**
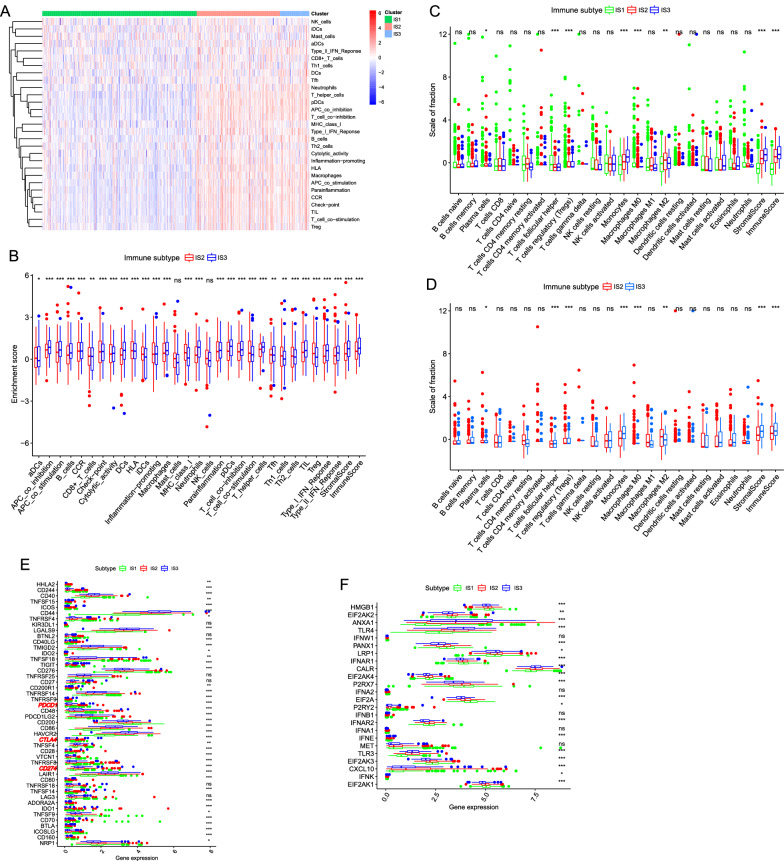
Features of TIME in different subtypes. **A** Based on the results of ssGSEA in LGG samples, the difference of enrichment score of each sample changes in IS1, IS2, and IS3, as the heatmap showed. **B** The difference of enrichment score of each sample changes in IS2 and IS2 shown in the boxplots. **C** The difference analysis of the abundance of immune cells and the level of the stromal, immune score on IS1, IS2, and IS3. **D** The difference analysis of the abundance of immune cells and the level of the stromal, immune score on IS2 and IS3. **E** The different expression levels of ICP genes in IS1, IS2, and IS3. **F** The different expression levels of ICD-related genes among three subtypes. *** p < 0.001, ** p < 0.01, * p < 0.05, ns: not significant

The ssGSEA score was employed for quantifying the activities or abundances of the immune signatures in the LGG samples. The enrichment scores (ES) in IS2 and IS3 were significantly higher than in the IS1 group in Fig. 5A. The difference analysis of ES between IS2 and IS3 indicated that in most cases, the samples in IS3 had higher enrichment scores and higher levels of immune infiltration (such as stromal score and immune score) than in the IS2 (Fig. 5B). CIBERSORT showed the proportions of different immune cells in 3 different subtypes (Fig. 5C). The proportion of 22 kinds of immune infiltrating cells was at a relatively low level, while the proportion in IS2 and IS3 was significantly higher than in IS1. Moreover, the boxplot (Fig. 5D) showed that M2 macrophages and T regulatory cells (Tregs) were the main components in IS2 than in other types, while monocytes and CD4^+^ T-helper cells in IS3 were significantly higher than in IS2. In addition, we investigated the expression level of 47 ICPs in different subgroups and found that 41 ICPs were differentially expressed among the immune subtypes (Fig. 5E). Moreover, CTLA4, PDCD1 (PD-1), and CD274 (PD-L1), as the primary immune checkpoints in cancers, had the highest expression level in IS2 and the lowest level in IS1 (p < 0.05, Fig. 5E). For immune cell deaths (ICDs), The expression level of 19 ICDs of all 24 kinds of ICDs were significantly different in three immune subtypes. The level of HMGB1, PANX1, IFNAR1, EIF2AK4, P2RX7, EIF2AK4, P2RX7, EIF2A, EIF2AK3, and EIF2AK1 were highest in IS1 than IS2 and IS3, EIF2AK2, LRP1, CALR, P2RX7, IFNAR2, MEF, and CXCL10 were overexpressed in IS2. While in IS3, the expression level of ANXA1, TLR4, and TLR3 were significantly up-regulated than in other subtypes (Fig. 5F). From what is mentioned above, we may conclude that IS1 was an "immune-desert phenotype", while IS2 indicating a potential immunosuppressive TIME and IS3 which may be related to an immunostimulatory characteristic TME were both immune "hot" type.

### Results of WGCNA

**Fig. 6:**
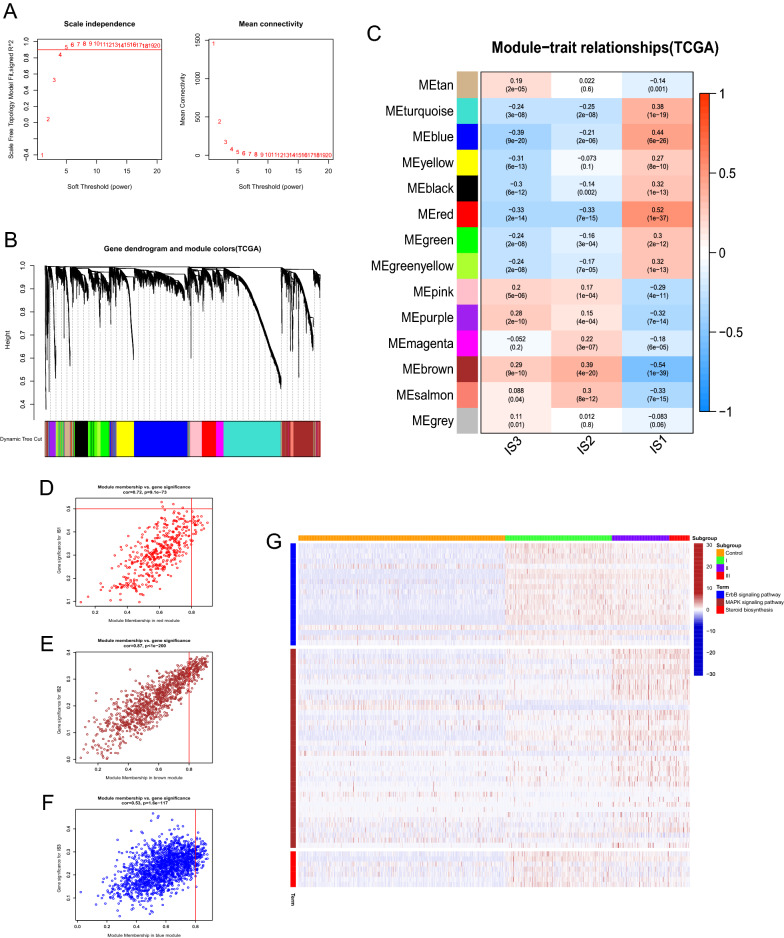
WGCNA of DEGs between different immune subtypes in TCGA-LGG. **A** Scale-free fit index and mean connectivity for various soft-thresholding powers (β). **B** DEGs were clustered using hierarchical clustering with a dynamic tree cut and merged based on a dissimilarity measure (1-TOM). **C** Relationship analysis between Traits and modules. Scatterplot of gene significance (GS. group) versus module membership (KME) for the **D** red, **E** brown, and **F** blue module. **G** Heatmap showed the activity of KEGG terms of blue, brown, and red module among non-tumor, IS1, IS2, and IS3 group, the deeper red color indicates the higher degree of KEGG terms enriched in each sample

Select five as the soft-thresholding power based on the scale-free fit index and the mean connectivity as Fig. 6A shown. Colors of dendrogram branches indicate different gene clusters, whereas the upper dendrogram shows sample clustering (Fig. 6B), 14 modules were screened out, and 3 modules and responding module genes were selected based on the relationship between modules and immune subtype. According to the correlation coefficient and p-value (Fig. 6C), the most relevant modules were red module (MEred) for IS1 (rho: 0.52, p < 0.05), brown module (MEbrown) for IS2 (rho:0.39, p < 0.05) and blue module (MEblue) for IS3 (rho: -0.39, p < 0.05). Figure 6D–F showed each module gene' 's module membership vs. gene significance scores, and genes with high module membership tended to have high gene significance in the scatter plots. KEGG terms enrichment analysis for module genes were performed (Fig. 7G), the genes of MEblue were mainly involved in the pathways of the ErbB signaling pathway, and genes in MEbrown were significantly related to the terms of MAPK signaling pathway, while genes of MEred were mainly participated in steroid biosynthesis.

## Discussion

Immunotherapy is a rapidly growing field, and tumor vaccines are a promising immunotherapeutic treatment modality in cancer research [28]. The ultimate goal of immunotherapy in cancer is eradicating tumors through vaccine strategies [29]. Through inducing anti-tumor immunity, a peptide vaccine targeting mutant IDH1 had been proved to be a feasible new strategy for the treatment of IDH1 (R132H) mutant gliomas in recent days [30, 31]. In this study, we identified four potential tumor antigens correlated to the immune infiltration level and screened out from mutant and up-regulated genes in LGG. Subsequently, the antigens’ association with prognosis and APCs were explored to assess their effectiveness and feasibility as antigens for mRNA tumor vaccines. Moreover, through the construction of robust immune subtypes, the characteristics of TIME and other clinical molecular characteristics of each subtype were investigated, and the population suitable for vaccination was identified on the basis of the immune landscape in three immune subtypes. Finally, the potential mechanisms and hub regulatory genes related to the immune subtype were then explored.

Tumor associated antigens (TAAs) are significantly over-expressed in cancer compared to normal cells [32]. Nowadays, advances in next-generation sequencing (NGS), bioinformatics and peptidomics have enabled the identification of non-synonymous mutations and other alterations of the cancer cell genome (intron retention, indels, frameshifts, etc.), emerging as neo-antigens and resulting in the development of personalized vaccines [33]. Neo-antigens could be recognized as non-self-epitopes and thereby enhance the immune reactivity against tumor cells [34].FCGBP (Fc fragment of IgG binding protein), a key regulator of TGF-1-induced epithelial-mesenchymal transition (EMT), was reported to be associated with the progression and prognosis of gallbladder cancer [35]. It reported that FLNC (filamin C) mutations cause myofibrillar myopathies [36], and it was also associated with central nervous system disease such as Friedreich's ataxia, fragile X syndrome, and spinocerebellar atrophy [37]. TLR7 (toll-like receptor 7) agonist MEDI9197 could modulate the tumor microenvironment leading to enhanced activity when combined with other immunotherapies [38]. Furthermore, study reported that CSF2RA (colony-stimulating factor 2 receptor) produced in the tumor was an essential factor affecting the progression and metastasis of breast cancer [39]. In this study, FCGBP, FLNC, TLR7, and CSF2RA were also correlated to the prognosis of LGG patients, which had not been reported before. Therefore, we considered these biomarkers with mutation possibility and up-regulated expression in LGG as potential TAAs, which provided a selection of tumor vaccine antigens and molecular targets of gliomas.

TIME plays a vital role in assisting anticancer vaccines to elicit therapeutically relevant tumor-specific immune responses [40]. The subtyping criteria developed for solid tumors could be well applied for the characterization of their immune microenvironment [41], Thorsson et al. identified six immune subtypes on the basis of a pan-cancer study in TCGA and revealed novel insights into the mechanisms and immunotherapy strategy across cancer types [42]. However, due to the existence of the blood–brain barrier and the specificity of TIME of gliomas, the immunotyping of pan-cancer maybe not suitable enough to distinguish the subtypes of glioma and provide a guideline for immunotherapy strategies. Based on the expression patterns of genes related to immune infiltration level in LGG, we divided glioma immune subtypes into IS1, IS2, and IS3, and defined them as immune desert type, immunosuppressive type, and immune promoting type, respectively. The three immune subtypes had distinct molecular, cellular, and clinical characteristics. In addition, we found that the patients in IS3 showed a better prognosis than other subtypes, which suggested immunotyping was a prognostic indicator in LGG. Base on the stemness of the tumor (mRNAsi), immunophenotyping could also be used to evaluate the ability of tumor progression and metastasis. As samples in IS1 were with higher value of mRNAsi, tumors of IS1 may be more likely to progress and metastasize. In addition to prognostic prediction, immunophenotyping could also predict the response and efficacy of mRNA vaccine therapy. IS1 with a poor correlation to immune infiltration level accounts for the vast majority of LGG, which indicated patients in IS1 receiving tumor vaccine treatment or immune checkpoint inhibitors (ICIs) therapy may not receive a better response or curative effect. Therefore, improving the infiltration level of tumor-killing immune cells is the precondition for ICIs of patients in IS1. Chemokines are necessary in transporting peripheral immune cells across the blood–brain barrier and activating these immune cells [43]. It may be a strategy to emphasize the critical role of chemokines in immune response for patients in IS1. Instead, patients in IS3 may be the most suitable candidates for tumor vaccination for its pro-inflammatory characteristics making mRNA cancer vaccine treatment more responsive and effective. However, as a subtype with moderate infiltration level and apparent immunosuppressive TIME, IS2 may lead to the difficulty in activating the activity of tumor-killing immune cells which played an anti-tumor role after receiving the mRNA tumor vaccine. Fortunately, the expression levels of PDCD1 (PD-1), CD274 (PD-L1), and CTLA4, the vital immune checkpoints in glioma, were significantly higher in IS2 than other subtypes, which indicated that patients receiving ICIs therapies might achieve a better curative effect [44]. As a result, combined with ICIs and mRNA tumor vaccine cold be an effective treatment strategy for patients with LGG in IS2.

Biomarkers of immune subtypes are the hub of linkage mechanism research, population screening, and typing specificity [45]. WGCNA revealed three key modules closely associated with each immune subtype and were of great significance to explore the potential biological mechanism of subtypes. KEGG and GO analysis showed that the red, brown, and blue modules had apparent differences in biology and involved pathways, which further suggested that the classification based on this study was of a high degree of discrimination.

## Conclusion

In conclusion, FCGBP, FLNC, TLR7, and CSF2RA are the potential antigens of the LGG mRNA vaccine which could be most beneficial for patients in IS3. It is crucial that this research provides a theoretical basis for mRNA vaccine against LGG, selects candidates suitable for cancer vaccination and provides a novel strategy of immunotherapy for LGG patients.

## Supplementary Information

**Additional file 1: Figure S1.** Diagrams summarizing mutation analysis in TCGA-LGG. (A) Summary of mutational signature analysis on 529 LGG samples. (B) Waterfall plot of the distribution of mutations. (C) Correlation analysis among the top 20 mutant genes in LGG samples.

## Data Availability

Publicly available datasets were analyzed in this study. This data can be found below: TCGA, https://www.cancer.gov/; GEPIA, http://gepia.cancer-pku.cn/detail.php; GTEx, https://www.gtexportal.org/home; TIMER, https://cistrome.shinyapps.io/timer/; STRING, https://string-db.org/cgi/input.pl.

## Abbreviations

LGG: Lower grade glioma
WHO: World Health Organization
TIME: Tumor immune microenvironment
TME: Tumor microenvironment
TCGA: The Cancer Genome Atlas
GTEx: Genotype-tissue expression
IRDEGs: Immune-related differentially expressed genes
GEPIA: Gene expression profiling interactive analysis
OS: Overall survival
DFS: Disease-free survival
TIMER: Tumor immune estimation resource
TIICs: Tumor immune infiltrating cells
APCs: Antigen-presenting cells
ssGSEA: Single-sample gene-set enrichment analysis
ICPs: Immune checkpoints
ICDs: Immunogenic cell death modulators
GO: Gene Ontology
KEGG: Kyoto encyclopedia of genes and genomes
Fig: Figure
IS1: Immune subtype 1
IS2: Immune subtype 2
IS3: Immune subtype 3
ICIs: Immune checkpoint inhibitors
WGCNA: Weight gene co-expression network analysis
