# Supplementary figures and images for: Identification of tumor antigens and immune subtypes in lower grade gliomas for mRNA vaccine development
Source: J Transl Med. 2021 Aug 17;19:352. doi: 10.1186/s12967-021-03014-x (PMC8369324; doi:10.1186/s12967-021-03014-x)

A

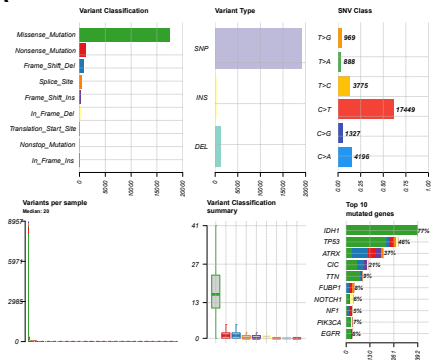

B

Altered in 488 (96.44%) of 506 samples.

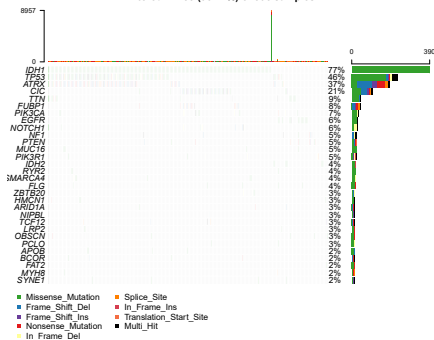

C

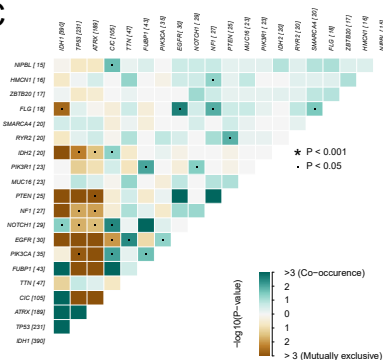

Supplement: Supplementary file 1 — Additional file 1: Figure S1. Diagrams summarizing mutation analysis in TCGA-LGG. (A) Summary of mutational signature analysis on 529 LGG samples. (B) Waterfall plot of the distribution of mutations. (C) Correlation analysis among the top 20 mutant genes in LGG samples. [file 12967_2021_3014_MOESM1_ESM.pdf]
